# Supplementary material for: Health-Promoting and Sustainable Behavior in University Students in Germany: A Cross-Sectional Study
Source: Int J Environ Res Public Health. 2023 Mar 23;20(7):5238. doi: 10.3390/ijerph20075238 (PMC10094390; doi:10.3390/ijerph20075238)
Supplement: Supplementary file 1 [file ijerph-20-05238-s001.zip › ijerph-2251969-supplementary.pdf]

Supplementary information

Article title: Health-promoting and sustainable behavior in university students in Germany: a cross-sectional study

Authors: Andrea Weber, Katharina Kroiss, Lydia Reismann, Petra Jansen, Gunther Hirschfelder, Anja M. Sedlmeier, Michael J. Stein, Patricia Bohmann, Michael F. Leitzmann, Carmen Jochem

Journal: International Journal of Environmental Research and Public Health

Questionary S1: Excerpt from the URStudisHealthSurvey questionnaire (translated from German into English)

The questionnaire was developed and administered to students at the University of Regensburg as part of a doctoral thesis (K.K.) on student health.

1: Sociodemographic data

I am a student at the University of Regensburg<br>*(obligatory)*	
	Yes
	No

What is your field of study?<br>*(obligatory)*	
	Catholic theology
	Law
	Economic sciences
	Medicine
	Philosophy, Arts, History, and Social sciences
	Computer and Data science
	Human sciences
	Linguistics, Literature, and Cultural studies
	Mathematics
	Physics
	Biology and Preclinical medicine
	Chemistry and Pharmacy

How long have you been studying at the University of Regensburg?<br>*(obligatory)*	
	1-2 semester(s)
	3-4 semesters
	5-6 semesters
	7-8 semesters
	9-10 semesters

	11-12 semesters
	>12 semesters

What gender do you identify with?<br>*(obligatory)*	
	Male
	Female
	Diverse
	No information

Please enter your age: … years<br>*(optional, free-text field)*
Providing your age is voluntary, but would be very helpful for scientific analyses.

How tall are you? … cm<br>*(optional, free-text field)*
Answering this question is voluntary, but it would be very helpful for the scientific analyses. The information about your height is not stored, it is only used to calculate the body mass index (BMI). You will see this displayed in the following.

What is your weight? … kg<br>*(optional, free-text field)*
Answering this question is voluntary, but it would be very helpful for the scientific analyses. The information about your weight is not stored, it is only used to calculate the BMI. You will see this displayed in the following.

Your BMI is: … kg/m²<br>*(displayed by LimeSurvey)*

2: Dietary habits

How important is healthy eating behavior to you?<br>*(obligatory)*	
	Very important
	Somewhat important

	Neither important nor unimportant
	Somewhat unimportant
	Not at all important

Please check the statements that apply to you in an ordinary week:<br>*(obligatory, Multiple choice)*	
	I eat a diversified diet and take advantage of food variety
	I eat at least three servings of vegetables and two servings of fruit daily
	I prefer whole grain products
	I eat milk and dairy products like yogurt and cheese daily
	I eat fish once or twice a week
	I eat no more than 300 to 600g of meat per week
	I prefer vegetable oils (e.g., rapeseed oil) and spreads produced from them
	I avoid hidden fats (these are often found in processed foods such as sausage, pastries, confectionery, fast food, and convenience foods)
	I use sugar in a sparse way and avoid sugar-sweetened foods and beverages
	I try to eat less or no salt
	I drink around 1.5 liters of water or other calorie-free drinks every day
	I make sure that my food is prepared gently
	I eat mindfully and take my time when eating
	I preferably shop regional, seasonal, or organic products
	I regularly cook at home
	None of the above
	No information

How would you rate the food offerings at the University of Regensburg*?
**Consider for example canteens, cafeterias, and kiosks at the University/University hospital of Regensburg*
(obligatory)

	Yes	Rather yes	Rather no	No	No opinion
I am satisfied with the quality of the food					
There are enough healthy options					
There are enough vegetarian/vegan options					

The value for money is realistic/accurate					
The offer is diverse enough					
I prefer to bring my own food from home					

3: Alcohol consumption, psychoactive substances and smoking

Do you drink alcohol?<br>*(obligatory)*	
	Yes
	No

(obligatory)			
	Yes	No	No information
Do you smoke?			

4: Physical activity and health promotion at the University of Regensburg

How many minutes do you do endurance-oriented exercise with moderate intensity in a usual week*?<br>*(optional, slide control for the unit minutes/week)*
**Examples are: fast walking, slow running (movement that is perceived as somewhat strenuous, where you can still talk but no longer sing)*<br>*Note: 5040 minutes = 84 hours, 2520 minutes = 42 hours, 2100 minutes = 35 hours, 1500 minutes = 25 hours, 600 minutes = 10 hours, 300 minutes = 5 hours.*

How many minutes do you do endurance-oriented exercise with high intensity in a usual week*?<br>*(optional, slide control for the unit minutes/week)*
**Examples are: running or fast cycling (movement that is perceived as strenuous, during which it is no longer possible to talk consistently)*<br>*Note: 5040 minutes = 84 hours, 2520 minutes = 42 hours, 2100 minutes = 35 hours, 1500 minutes = 25 hours, 600 minutes = 10 hours, 300 minutes = 5 hours.*

On how many days do you engage in muscle-strengthening physical activities in a usual week*?<br>*(optional, slide control for the unit days/week)*
**Examples are: Workouts with your own body weight, strength training using equipment.*

How many hours do you spend sitting on an ordinary day?<br>*(optional, slide control for the unit hours/day)*

How many hours of the above* do you spend sitting at the university each day?<br>*(optional, slide control for the unit %)*
**Please note: The information is given in percentage.*

I avoid long, uninterrupted periods of sitting and take short breaks every 30 minutes for movement/exercise.<br>*(obligatory)*	
	Yes
	Rather yes
	Rather no
	No

In the summer term, I usually get to the university by<br>*(obligatory)*	
	Public transport
	Foot
	My own car/vehicle
	The bike/electric bike
	No information

You stated that you regularly cycle to university during summer term. How long does it take you to get **to university and back** during summer term?<br>*(optional, slide control for the unit minutes/day)*

In the winter term, I usually get to the university by<br>*(obligatory)*	
	Public transport
	Foot

	My own car/vehicle
	The bike/electric bike
	No information

You stated that you regularly cycle to university during winter term. How long does it take you to get **to university and back** during winter term? *(optional, slide control for the unit minutes/day)*

Which of the following health promotion topics at the University of Regensburg would you be interested in? *(obligatory, Multiple choice)*	
	Relaxation/ Stress management
	Mindfulness
	Dietary habits
	Smoking cessation
	Physical education and exercise programs
	General health counselling
	Health diagnostics for safe and targeted training
	Cooking courses
	Other
	None of the above

You selected „other“ in the previous question. Please use the following text field* for specific comments and requests: *(optional, free-text field)*
**Please make sure that you do not enter any information in the free-text field that could lead to a conclusion about your person.*

7: Global health und sustainable behavior

Which health consequences of climate change have you already heard about? *(obligatory, Multiple choice)*	
	Heatstroke/Heat stress
	Cardiovascular issues
	Increased allergies

	Mental health issues (e.g., due to natural disasters)
	Respiratory symptoms (e.g., due to air pollution)
	Increasing global malnutrition
	Spreading of infectious diseases
	I am well informed about these and other health consequences of climate change
	I am not aware that climate change can have negative impacts on health

Do you actively try to reduce your carbon footprint (that means your climate affecting emissions) in your everyday life? *(obligatory)*	
	Yes, a lot
	Yes, mostly
	Neither nor
	Unlikely
	Not at all

How likely would you consider yourself to engage in the following? *(obligatory)*					
	Very likely	Likely	Neither nor	Unlikely	Very unlikely
Ride a bicycle /electric-bike instead of a fuel-consuming car in your everyday life					
Use public transportation (e.g., bus, train) for travelling instead of cars or planes					
Regularly consume a diet predominantly free of meat and fish products (i.e., a vegetarian diet)					
Regularly consume a diet entirely free of animal products (i.e., a vegan diet)					

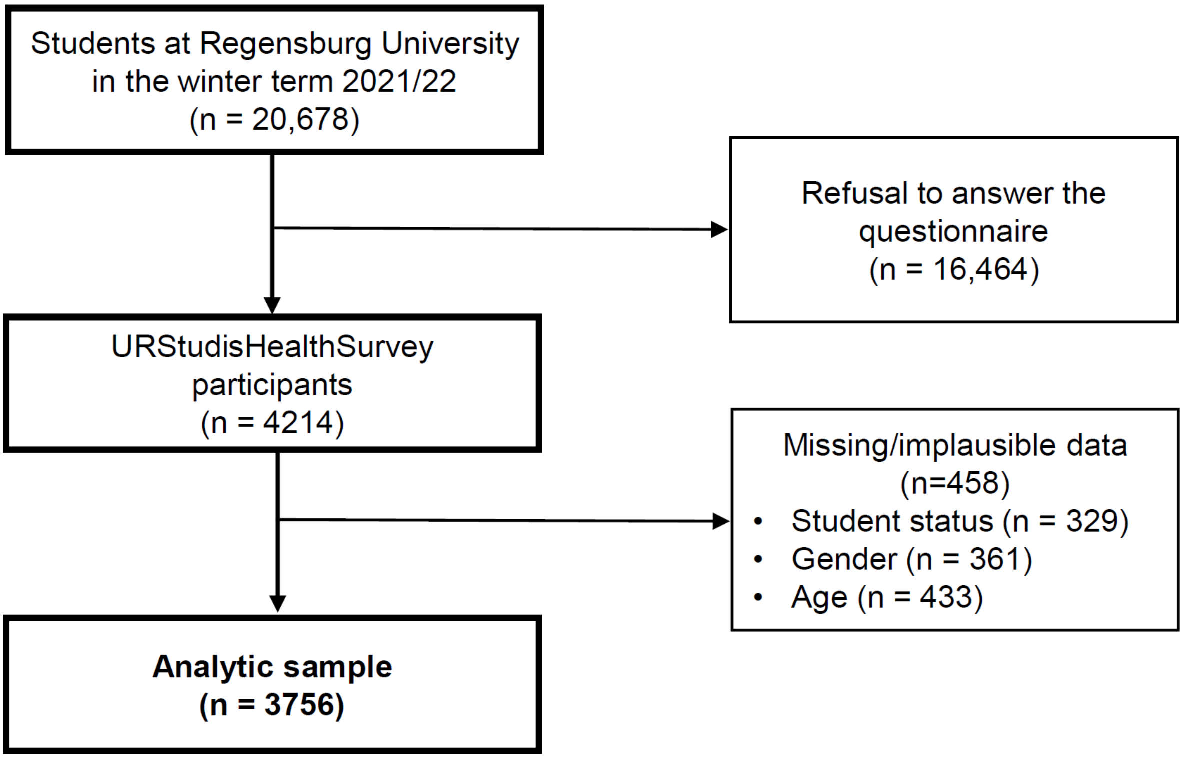

Figure S1: Flow chart

Table S1: Informedness by age and gender: What health consequences of climate change have you already heard about?

Gender	Age group	Heatstroke/ Heat stress	Cardiovascular issues	Increased allergies	Mental health issues	Respiratory symptoms	Global malnutrition	Infectious diseases	Well informed about all these	Not aware about health impacts of climate change
Men	< 22	62	51	52	57	67	80	70	31	11
	22-25	59	56	55	57	65	74	68	34	11
	> 25	72	66	63	63	72	78	73	44	9
Women	< 22	50	46	59	64	67	78	72	28	10
	22-25	57	51	61	61	68	80	77	30	9
	> 25	63	63	62	62	69	76	72	38	9

Proportion of affirmative answers; n=3021

32 participants reporting diverse gender were excluded from this analysis to protect participant anonymity.

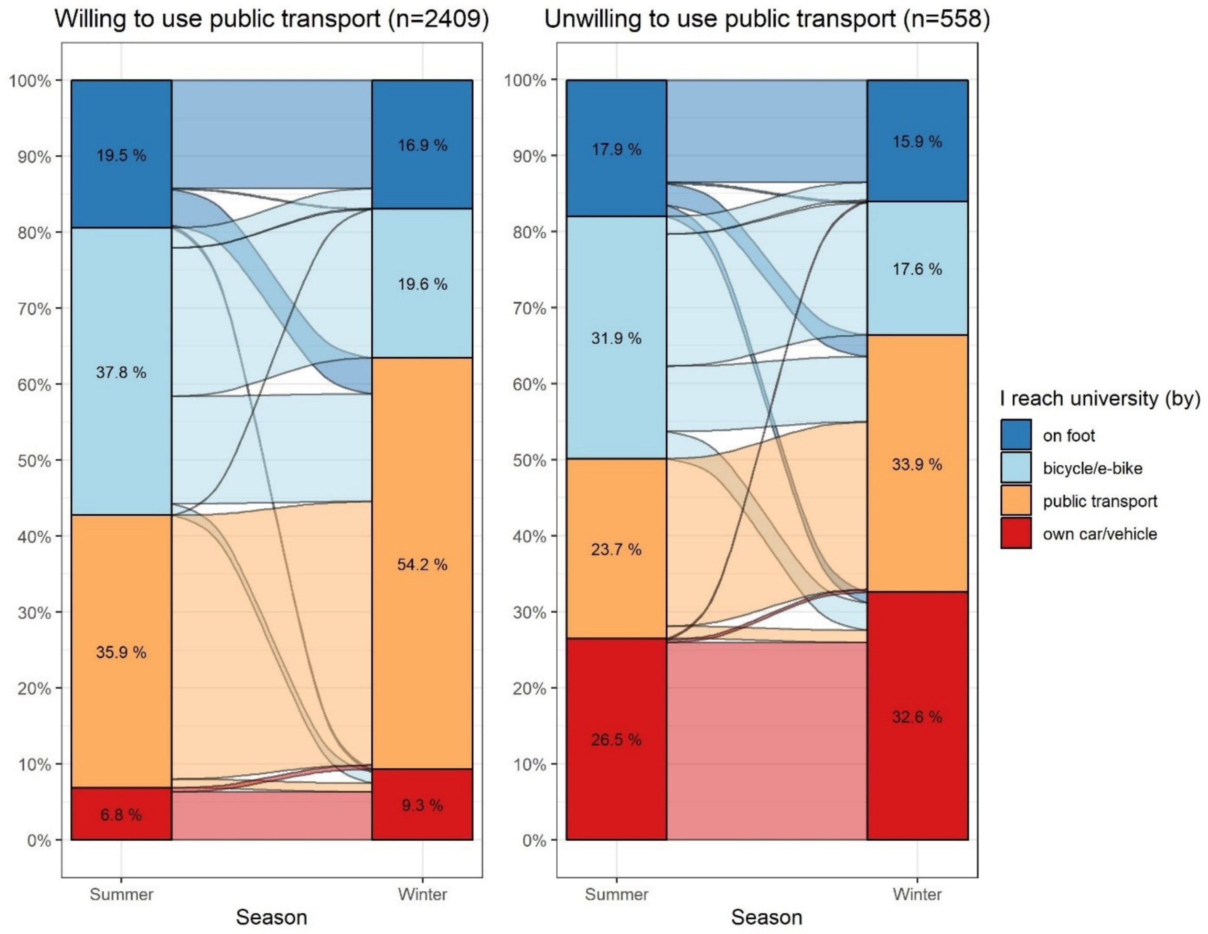

Figure S2: Means of transport commuting to and from university in the summer and the winter stratified by willingness to use public transport (n=2967).

The curves between the bar charts represent changes in the composition of the strata between summer and winter.

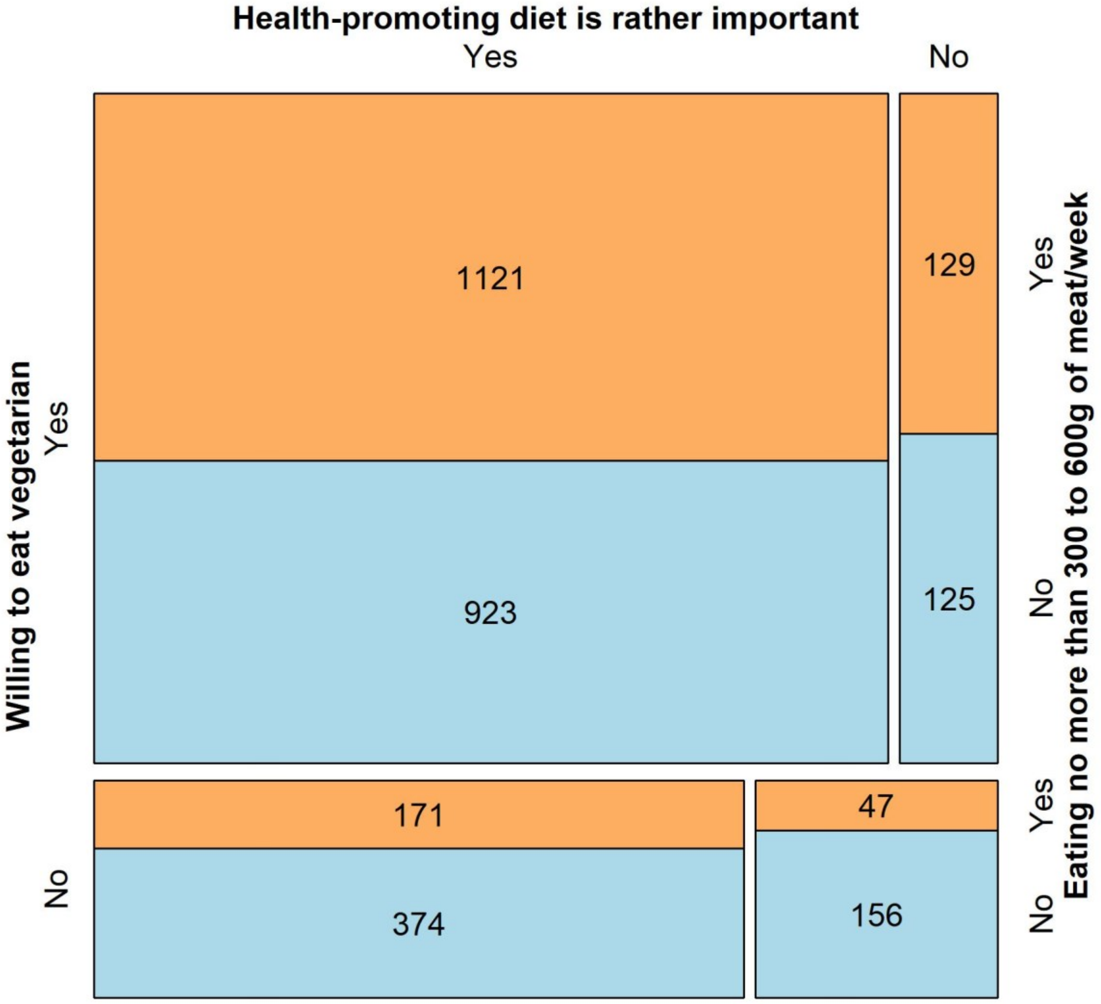

Figure S3: Relations between willingness to eat a vegetarian diet, meat consumption and personal value of a health-promoting diet (n=3046).
